# Review of Various Clinical Assessment Indices and Orthodontic Management for Temporomandibular Joint Disorders

**DOI:** 10.7759/cureus.30492

**Published:** 2022-10-19

**Authors:** Nikhil Kumar, Pallavi Daigavane, Shraddha Jain, Namrata Mantri

**Affiliations:** 1 Department of Orthodontics, Sharad Pawar Dental College, Datta Meghe Institute of Medical Sciences, Wardha, IND; 2 Department of Otorhinolaryngology, Jawaharlal Nehru Medical College, Datta Meghe Institute of Medical Sciences, Wardha, IND; 3 Department of Public Health Dentistry, Sharad Pawar Dental College, Datta Meghe Institute of Medical Sciences, Wardha, IND

**Keywords:** mfiq, temporomandibular disorders, fonseca index, craniomandibular index, helkimo index

## Abstract

The term "temporomandibular disorders" (TMDs) refers to a variety of problems involving the muscles of the masticatory system and the jaw. The most common symptoms of TMD are pain in the face, headaches, clicking or popping in the joints, and difficulties with jaw function. The severity of TMD can be measured with a number of different scales, including the Helkimo, Craniomandibular Index (CMI), Mandibular Functional Impairment Questionnaire (MFIQ), Fonseca scale and Diagnostic Criteria for Temporomandibular Disorders (DC/TMD) scales. The former focuses on the patient's chief complaint, while the latter takes into account secondary symptoms such as limited mobility, impaired temporomandibular joint (TMJ) function, muscle pain, and discomfort during mandibular motion. According to the severity of the issue, the results can be used to categorise the situation. To effectively treat TMD, one must first determine their index score and then formulate a treatment strategy based on that score.

## Introduction and background

The mandible and the temporal bone of the skull articulate at the temporomandibular joint (TMJ). It is referred to as a ginglymoarthrodial joint because it allows for gliding motion in one plane while also allowing for hinging motion in another, earning it the name "ginglymoid joint" [1].

The term "temporomandibular disorders" (TMD) refers to a set of musculoskeletal problems that affect the masticatory muscles, TMJ, and related structures and are characterized by discomfort and/or dysfunction [2]. TMD's etiology is typically seen as complex. Untreated malocclusions, unstable occlusal patterns, psychological stress, trauma, genetic susceptibility, and structural issues have all been proposed as potential causative factors [3]. Different symptoms and indicators are found in TMD. Facial pain, headaches, joint sounds, and problems with jaw function are the most typical TMD symptoms. Patients with TMD may also exhibit a variety of symptoms, including discomfort, restricted range of motion, motion deviation, and joint noise. As diagnostic criteria and outcome measures, these symptoms' dependability, validity, and accuracy have not yet been fully established. A clinician has a wide range of resources at their disposal for evaluating TMD, including indices, questionnaires, protocols, scales, and diagnostic criteria. Indices are composed of structured forms for grading the intensity of symptoms and assigning scores to signs. As a result, indices can be used to accurately quantify the signs and symptoms of various illnesses using symptomatology and a standardized clinical examination [4].

The mainstay is the treatment planning for orthodontic treatment is made simple with the use of these indices for TMD in many categories. Analgesics, heat fomentation, joint rest, and orthodontic management are examples of conservative treatments for acute instances. The clinical assessment indices provide a severity score that aids in determining whether orthodontic treatment is necessary.

## Review

Clinical assessment indices

The two that are most frequently used are those presented by Helkimo [4] and by Fricton and Schiffman [5]. The anamnestic index and the clinical dysfunction index are both included in the Helkimo index. The former is based mostly on the primary complaint, whereas the latter examines factors including reduced range of motion, decreased TMJ function, muscular pain, TMJ pain, and pain with mandibular movement. There are three scoring categories for each of these items. Lastly, the results can be used to classify the problem according to how bad it is.

Helkimo Index

The process of obtaining Helkimo's indices [4] began with the anamnestic dysfunction index (Ai), which is made up of the following classes: Ai0 indicates the absence of any discernible dysfunctional symptoms; AiI indicates minor symptoms such as TMJ creasing and clicking noises, as well as jaw tightness or fatigue; and AiII indicates significant dysfunctional symptoms such as pain when moving the face and jaw; locking; difficulty opening the mouth wide; and locations.

After the clinical exam, the following classes of the clinical index of dysfunction (Di) were found based on the results of an objective assessment of the TMJ, masticatory, and cervical muscles: (1) Normal, that is, mandibular range of motion is 40 mm, horizontal movements (7 mm = 0 points, mildly impaired = 1 point, and severely impaired = 5 points). (2) Impaired TMJ function: TMJ noises in one or both joints and/or deviation (42 mm = 1 point, locking and/or luxation of the TMJ = 5 points); smooth movements without TMJ noises, evaluated with auscultation and deviation; and applying a rule to opening or closing movements. (3) To rate the severity of TMJ pain, the degree to which palpation of the masticatory muscles is painful ranges from 0 (no tenderness) to 5 (extreme tenderness). Origins and insertions of the masseter, posterior and middle temporalis, anterior temporalis, posterior mandible, sub mandible, lateral pterygoid, and temporalis tendons were all palpable. Pain felt in a different area of the body as a result of an injury to a nearby muscle was taken as a good sign. (4) Any tenderness to palpation of the masticatory muscles is worth 1 point, tenderness in two to three locations is worth 3 points, and tenderness in four or more locations is worth 5 points when rating TMJ pain. Several of the facial muscles, including the masseter, the posterior and middle temporalis, the anterior temporalis, the posterior mandibular region, the submandibular region, the lateral pterygoid area, and the temporalis tendon, were all palpable. Soreness elsewhere in the muscle that causes referred pain was taken as an indication that the problem lay in the targeted area. (5) Mandibular pain is scored as follows: 0 points for no pain, 1 point for mild pain on one movement, and 5 points for severe pain on two or more movements [5].

The totals of the points associated with these data allowed for the following categorizations to be drawn: Di0 = 0 Value (dysfunction group 0) displaying no signs or symptoms detectable by medical professionals. DiI = 1-4 (mild dysfunction, group 1) category. Moderate dysfunction corresponds to a DiII score between 5 and 9 points, which corresponds to dysfunction group 2. DiIII = 10-13 points (dysfunction group 3), 15-17 points (dysfunction group 4), and 20-25 points (dysfunction group 5), all of which indicate extremely severe dysfunction [4].

Craniomandibular Index (CMI)

Every positive item earns 1 point toward the Craniomandibular Index (CMI) [4,5], while every negative item earns 0 points. Positive comments were used to evaluate the following seven key factors:

Considerations for mandibular motions (MM) included the following: (1) The patient's maximum mouth opening is measured from the midpoint of the upper incisor to the midpoint of the lower incisor. This item fared best when either 40 mm or 60 mm in size. The range of motion estimates was made without considering the overbite values. (2) Passive stretch opening: the maximum measurement reached by the examiner after the patient has opened maximally voluntarily. When the measurement was 42 mm or greater, the item received a favourable score. (3) The maximum opening size must be less than 40 mm for this item to be advantageous. (4) Achy while opening. When there was pain at the maximal opening, this item was beneficial. (5) Opening for jerky. Even though the opening wasn't smooth or constant, this item was still positive. (6) An s-curve on the opening or closing: a variation from the straight line. When the mandible's midline was 42 mm away from the upper jaw's midline, this item was positive. (7) Lateral deviation at maximum aperture. This item was favourable when positioned 42 millimetres off the midline. (8) Discomfort caused by a protrusion. The product helped alleviate any pain felt at the peak of protrusion. (9) No further sticking out is allowed. When the maxillary incisor labial surface and mandibular incisor labial surface were measured, the maximum voluntary protrusion was 57 mm, and the overjet value was added, the item was positive. (10) Right-sided pain upon protrusion. (11) Restrictions on the right side of the body prevent full laterotrusion. This was effective at 57 mm. The maximum extent of both mandibular lateral motions was determined by measuring the gap between the midline of the maxillary incisors and a vertical line engraved on the mandibular incisors at the position where the former falls in occlusion. (12) A left laterotrusion that hurts. restriction on left laterotrusion. (13) When 57 mm, this thing was good. (14) Clinical locking on opening is when the condylar head is dislocated forward out of the glenoid fossa and then fixed in that position without a time limit. (15) Clinical lockup on closing: a temporary or permanent impediment to translation during the opening. (16) Jaw rigidity after manipulation. If the jaw could not be manually rotated, whether voluntarily or involuntarily, this item was positive [5].

The following elements for TMJ noise (TN) were considered: reproducible opening click: a sound made each time a door is opened; repeatable closing click: the sound made each time a door closes; reciprocal click: a sound made each time a door opens and closes; replicable laterotrusive click: sound produced during each laterotrusive motion; a noise that is occasionally present during movement but is not usually reproducible; a thin grating sound known as crepitus; a coarse grating sound known as crepitus; popping: an opening sound that is loud and audible from a distance [4]. Muscle and TMJ palpation consisted of the extraoral and intraoral palpation of the jaw muscles, the palpation of the neck muscles, and the palpation of the temporomandibular joints (TMJ). We have talked about how to use pressure, how to calibrate it, how long to apply it, and whether or not it will cause referred pain.

Extraoral palpation (EP) of the jaw muscles could be done on the anterior temporalis, the deep temporalis, the middle temporalis, the deep masseter, the anterior masseter, the inferior masseter, the posterior digastric, the medial pterygoid, and the vertex. palpating the jaw muscles involved in the lateral pterygoid area, the medial pterygoid, and the temporalis insertion (IP) was difficult.

Palpation of the neck muscles included the supraspinatus, mediospinatus, and infraspinatus, the trapezius insertion, the upper trapezius, and the spleniuscapitis (NP). When palpating the TMJ, the lateral capsule, posterior capsule, and superior capsule were all taken into account (TP).

Because they aren't employed in the CMI, assessments of structural instability and occlusal instability weren't included [5]. The CMI was developed to assign the DI and PI equal weight and ratings ranging from 0 to 1. This was accomplished by dividing the total number of items by the sum of the affirmative responses to the MM, TN, and TP = 26. By dividing the total number of items by the sum of the positive reactions to palpation of the jaw and neck muscles (EP + IP + NP), the PI was determined = 36. CMI is calculated by dividing the sum of DI and PI by 2.

Mandibular Function Impairment Questionnaire (MFIQ)

Assessment of anatomical and physiological factors crucial to diagnosis constitutes the initial step. The patient's sense of functional impairment is the focus of the second part of the functional assessment. Jaw movement restriction evaluation. Digital palpation was used to feel the lateral aspect of the temporomandibular joint at maximum protrusion, then maximum opening from the maximum protruded position. The following criteria were used to grade joint mobility on a 5-point scale [6]: -2 = No or barely perceptible translation during protrusive motion or opening after peak protrusion. -1 = Translation felt during protrusion; minimal extra translation during opening following maximum protrusion; 0 = Additional translation is apparent during opening following the maximum protrusive movement, and translation is felt during protrusive movement. 1 = Protrusive movement causes translation that is easily felt; excessive extra translation is felt during opening after the most protrusive action. 2 = Translation is very noticeable during protrusive movement, and excessive extra translation is very noticeable during opening following the most protrusive movement.

Assessment of the Patient's Appreciation of Function Impairment: In the clinical environment, a preliminary questionnaire is utilised to get patients' subjective opinions on several items meant to gauge functional capacities. Seventeen items make up the Mandibular Function Impairment Questionnaire (MFIQ) (Tables 1-4).

**Table 1:** Mandibular Function Impairment Questionnaire.

Questionnaire	Possible answers
Due to the complaints about your jaw, how much difficulty do you have with:	Item score (0-4):i No difficulty-0 A little difficulty -1 Quite a bit of difficult -2 Much difficulty -3 Very difficult or impossible without help-4
1. Social activities	
2. Speaking	
3. Taking a large bite	
4. Chewing hard food	
5. Chewing soft food	
6. Work and/or daily activities	
7. Drinking	
8. Laughing	
9. Chewing resistant food	
10. Yawning	
11. Kissing	
Eating food includes taking a bite, chewing, and swallowing. How much difficulty do you have with eating:	
12. A hard cookie	
13. Meal	
14. A raw carrot	
15. French bread	
16. Peanuts/almonds	
17. An apple	

**Table 2:** Calculation of raw component score

Item score	i	range 0-4
Numbers of items	N	
Sum item scares	S = i,+...+i_N_	range 0-4 N
Raw Component Score	C = S/4N	range 0-1

**Table 3:** Calculation of level of function impairment i : item score C : Raw Component Score

Rule for i	Rule for C	Function impairment rating scale: firs
all i < 2	C ≤ 0.3	0
at least one i > 2	C ≤ 0.3	1
all i < 3	0.3 < C ≤ 0.6	2
at least one i > 3	0.3 < C ≤ 0.6	3
all i ≠ 4	C > 0.6	4
at least one i = 4	C > 0.6	5

**Table 4:** Qualitative level of function impairment

Grading	Value
I Low	0 or 1
II Moderate	2 or 3
III Severe	4 or 5

The patient is given a 5-point Likert scale for each item so they can rate how difficult it is to complete a specific mandibular task. The difficulty scale goes from 0 (no difficulty) through 1 (a little difficulty), 2 (quite a bit of difficulty), 3 (a lot of trouble), and 4 (very tough or impossible without aid) [6].

Fonseca Anamnestic Index

In 1994, the Fonseca Anamnestic Index (FAI), a method for assessing the severity of TMD based on its symptoms and indications, was put out. This questionnaire, which was based on the Helkimo Anamnestic Index, has a good positive correlation and reliability rating (95%) compared to the latter. Ten questions make up the exam, which categorises patients into four categories based on the severity of their symptoms: (a) without TMD, (b) mild TMD, (c) moderate TMD, and (d) severe TMD [7,8]. The following are some of the inquiries: 1) Is it tough for you to open your mouth wide? 2) Do you have trouble opening your mouth wide? 3) Do you experience muscle soreness or tiredness during chewing? 4) Do you experience headaches frequently? 5) Do you suffer from neck discomfort or a stiff neck? 6) Do you experience ear pain or temporomandibular joint pain? 7) Have you ever heard any noise in your temporomandibular joint while you were eating or opening your mouth? 8) Do you have any bad habits, such as clenching or grinding your teeth? 9) Do you think your teeth don't fit together well? 10) Do you consider yourself to be an anxious (tense) person? [8]. The three possible responses to each question are "Yes" (10 points), "Sometimes" (5 points), and "No" (0 points). Following the addition of the responses, the patients are categorised using the following values: 0 to 15 (without TMD); 20 to 40 (mild TMD); 45 to 65 (moderate TMD); and 70 to 100 (severe TMD).

As there are no criteria to attain a numeric value to decide the severity of TMD, indices play an important role to determine the prevalence of this disorder in a specified population. Helkimo was considered a pioneer in developing an index to measure the severity and pain in TMD patients, whereas the CMI index was developed to provide a standardized measure of the severity of problems in mandibular movement, TMJ noise, and muscle and joint tenderness. It was designed to have clearly defined objective criteria, simple clinical methods, and ease of scoring; it is divided into the Dysfunction Index and the Palpation Index.

Recent Advances in the Diagnosis of TMDs

For the first time, the introduction of the Research Diagnostic Criteria for Temporomandibular Disorders (RDC/TMD) criteria allowed researchers to be positive that they were picking samples with a reliable and valid diagnostic instrument. However, due to an unfortunate reputation of being complex and time-consuming, the RDC/TMD has not been part of standard clinical practise. Recent efforts have been made to simplify and shorten the RDC/TMD criteria for active clinical practise. The newly recommended Diagnostic Criteria for TMD (DC/TMD) protocol includes both a valid screener for detecting any pain-related TMD as well as valid diagnostic criteria for differentiating the most common pain-related TMD (Table 5) and for one intra-articular disorder (Table 6) [9,10].

**Table 5:** Diagnostic Criteria for the Most Common Pain-Related Temporomandibular Disorders The time frame for assessing pain including headache is in “the last 30 days” since the stated sensitivity and specificity of these criteria were established using this time frame. Although the specific time frame can be dependent on the context in which the pain complaint is being assessed, the validity of this diagnosis based on different time frames has not been established. †The examiner must identify with the patient all anatomical locations that they have experienced pain in the last 30 days. For a given diagnosis, the location of pain induced by the specified provocation test(s) must be in an anatomical structure consistent with that diagnosis. ‡“Familiar pain” or “familiar headache” is based on patient report that the pain induced by the specified provocation test(s) has replicated the pain that the patient has experienced in the time frame of interest, which is usually the last 30 days. “Familiar pain” is pain that is similar or like the patient’s pain complaint. “Familiar headache” is pain that is similar or like the patient’s headache complaint.

	Indicated history and exam criteria must be met for each diagnosis. Myalgia
Description	Pain of muscle origin that is affected by jaw movement, function, or parafunction, and replication of this pain occurs with provocation testing of the masticatory muscles.
HISTORY	Positive for both of the following: Pain in the jaw, temple, in the ear, or in front of ear; AND Pain modified with jaw movement, function or parafunction.
Examination	Positive for both of the following: Confirmation of pain location(s) in the temporalis or masseter muscle(s); AND Report of familiar pain in the temporalis or masseter muscle(s) with at least one of the following provocation tests: Palpation of the temporalis or masseter muscle(s); OR Maximum unassisted or assisted opening movement(s)
Validity	Sensitivity 0.90; Specificity 0.99
Comments	The pain is not better accounted for by another pain diagnosis. Other masticatory muscles may be examined as dictated by clinical circumstances, but the sensitivity and specificity for this diagnosis based on these findings have not been established.
	Types of myalgia as differentiated by provocation testing with palpation: Local myalgia, myofascial pain and myofascial pain with referral
	Local myalgia
Description	Pain of muscle origin as described for myalgia with localization of pain only at the site of palpation when using the myofascial examination protocol
HISTORY	Positive for both of the following: Pain in the jaw, temple, in the ear, or in front of ear; AND Pain modified with jaw movement, function or parafunction
Examination	Positive for all of the following: Confirmation of pain location(s) in the temporalis or masseter muscle(s); AND Report of familiar pain with palpation of the temporalis or masseter muscle(s); AND Report of pain localized to the site of palpation.
Validity	Sensitivity and specificity have not been established.
Comments	The pain is not better accounted for by another pain diagnosis. Other masticatory muscles may be examined as dictated by clinical circumstances but the sensitivity and specificity for this diagnosis based on these findings have not been established.
	Myofascial pain
Description	Pain of muscle origin as described for myalgia with pain spreading beyond the site of palpation but within the boundary of the muscle when using the myofascial examination
History	Positive for both of the following: 1. Pain** in the jaw, temple, in the ear, or in front of ear; AND 2. Pain modified with jaw movement, function or parafunction.
Exam	Positive for all of the following: 1. Confirmation† of pain location(s) in the temporalis or masseter muscle(s); AND 2. Report of familiar pain‡ with palpation of the temporalis or masseter muscle(s); AND 3. Report of pain spreading beyond the site of palpation but within the boundary of the muscle.
Validity	Sensitivity and specificity have not been established.
Comments	The pain is not better accounted for by another pain diagnosis. Other masticatory muscles may be examined as dictated by clinical circumstances but the sensitivity and specificity for this diagnosis based on these findings have not been established.
	Myofascial pain with referral
Description .	Pain of muscle origin as described for myalgia with referral of pain beyond the boundary of the muscle being palpated when using the myofascial examination protocol. Spreading pain may also be present
History	Positive for both of the following: 1. Pain** in the jaw, temple, ear, or in front of ear; AND 2. Pain modified with jaw movement, function, or parafunction.
Exam	Positive for all of the following: 1. Confirmation† of pain location(s) in the temporalis or masseter muscle(s); AND 2. Report of familiar pain‡ with palpation of the temporalis or masseter muscle(s); AND 3. Report of pain at a site beyond the boundary of the muscle being palpated.
Validity	Sensitivity 0.86; Specificity 0.98
Comments	The pain is not better accounted for by another pain diagnosis. Other masticatory muscles may be examined as dictated by clinical circumstances but the sensitivity and specificity for this diagnosis based on these findings have not been established.
	Arthralgia
Description	Pain of joint origin that is affected by jaw movement, function, or parafunction, and replication of this pain occurs with provocation testing of the TMJ.
History	Positive for both of the following: 1. Pain** in the jaw, temple, ear, or in front of ear; AND 2. Pain modified with jaw movement, function, or parafunction.
Exam	Positive for both of the following: 1. Confirmation† of pain location in the area of the TMJ(s); AND 2. Report of familiar pain‡ in the TMJ with at least one of the following provocation tests: a. Palpation of the lateral pole or around the lateral pole; OR b. Maximum unassisted or assisted opening, right or left lateral, or protrusive movement(s).
Validity	Sensitivity 0.89; Specificity 0.98
Comments	The pain is not better accounted for by another pain diagnosis.
	Headache attributed to TMD
Description	Headache in the temple area secondary to pain-related TMD (see Note) that is affected by jaw movement, function, or parafunction, and replication of this headache occurs with provocation testing of the masticatory system.
History	Positive for both of the following: 1. Headache** of any type in the temple; AND 2. Headache modified with jaw movement, function, or parafunction.
Exam	Positive for both of the following: 1. Confirmation† of headache location in the area of the temporalis muscle(s); AND 2. Report of familiar headache‡ in the temple area with at least one of the following provocation tests: a. Palpation of the temporalis muscle(s); OR b. Maximum unassisted or assisted opening, right or left lateral, or protrusive movement(s).
Validity	Sensitivity 0.89; Specificity 0.98
Comments	The headache is not better accounted for by another headache diagnosis.

**Table 6:** Diagnostic Criteria for the Most Common Intra-articular Temporomandibular Disorders The time frame for assessing selected biomechanical intra-articular disorders is in “the last 30 days” since the stated sensitivity and specificity of these criteria were established using this time frame. Although the specific time frame can be dependent on the context in which the noise or biomechanical complaints are being assessed, the validity of this diagnosis based on different time frames has not been established.

	Indicated history and exam criteria must be met for each diagnosis except subluxation, which is based only on history.
	Disc displacement with reduction
Description	An intracapsular biomechanical disorder involving the condyle-disc complex. In the closed mouth position, the disc is in an anterior position relative to the condylar head and the disc reduces upon opening of the mouth. Medial and lateral displacement of the disc may also be present. Clicking, popping, or snapping noises may occur with disc reduction. A history of prior locking in the closed position coupled with interference in mastication precludes this diagnosis.
History	Positive for at least one of the following: 1. In the last 30 days,** any TMJ noise(s) present with jaw movement or function; OR 2. Patient report of any noise present during the exam.
Exam	Positive for at least one of the following: 1. Clicking, popping, and/or snapping noise during both opening and closing movements, detected with palpation during at least one of three repetitions of jaw opening and closing movements; OR 2a. Clicking, popping, and/or snapping noise detected with palpation during at least one of three repetitions of opening or closing movement(s); AND 2b. Clicking, popping, and/or snapping noise detected with palpation during at least one of three repetitions of right or left lateral, or protrusive movement(s).
Validity	Without imaging: sensitivity 0.34; specificity 0.92. Imaging is the reference standard for this diagnosis.
Imaging	When this diagnosis needs to be confirmed, TMJ MRI criteria2 are positive for both of the following: 1. In the maximum intercuspal position, the posterior band of the disc is located anterior to the 11:30 position and the intermediate zone of the disc is anterior to the condylar head; AND 2. On full opening, the intermediate zone of the disc is located between the condylar head and the articular eminence.
	Disc displacement with reduction with intermittent locking
Description	An intracapsular biomechanical disorder involving the condyle-disc complex. In the closed mouth position, the disc is in an anterior position relative to the condylar head, and the disc intermittently reduces with opening of the mouth. When the disc does not reduce with opening of the mouth, intermittent limited mandibular opening occurs. When limited opening occurs, a maneuver may be needed to unlock the TMJ. Medial and lateral displacement of the disc may also be present. Clicking, popping, or snapping noises may occur with disc reduction.
History	Positive for both of the following: 1a. In the last 30 days,** any TMJ noise(s) present with jaw movement or function; OR 1b. Patient report of any noise present during the exam; AND 2. In the last 30 days,** jaw locks with limited mouth opening, even for a moment, and then unlocks.
Exam	Positive for at least one of the following: 1. Clicking, popping, and/or snapping noise detected during both opening and closing movements, detected with palpation during at least one of three repetitions of jaw opening and closing movements; OR 2a. Clicking, popping, and/or snapping noise detected with palpation during at least one of three repetitions of opening or closing movement(s); AND 2b. Clicking, popping, and/or snapping noise detected with palpation during at least one of three repetitions of right or left lateral, or protrusive movement(s).
Validity	Without imaging: sensitivity 0.38; specificity 0.98. Imaging is the reference standard for this diagnosis.
Imaging	When this diagnosis needs to be confirmed, the imaging criteria are the same as for disc displacement with reduction if intermittent locking is not present at the time of imaging. If locking occurs during imaging, an imaging- based diagnosis of disc displacement without reduction will be rendered and clinical confirmation of reversion to intermittent locking is needed.
	Disc displacement without reduction with limited opening
Description	An intracapsular biomechanical disorder involving the condyle-disc complex. In the closed mouth position, the disc is in an anterior position relative to the condylar head, and the disc does not reduce with opening of the mouth. Medial and lateral displacement of the disc may also be present. This disorder is associated with persistent limited mandibular opening that does not reduce with the clinician or patient performing a manipulative maneuver. This is also referred to as “closed lock.” This disorder is associated with limited mandibular opening.
History	Positive for both of the following: 1. Jaw locked so that the mouth would not open all the way; AND 2. Limitation in jaw opening severe enough to limit jaw opening and interfere with ability to eat.
Exam	Positive for the following: 1. Maximum assisted opening (passive stretch) movement including vertical incisal overlap < 40 mm.
Validity	Without imaging: sensitivity 0.80; specificity 0.97. Imaging is the reference standard for this diagnosis
Imaging	When this diagnosis needs to be confirmed, TMJ MRI criteria are positive for both of the following: 1. In the maximum intercuspal position, the posterior band of the disc is located anterior to the 11:30 position and the intermediate zone of the disc is anterior to the condylar head, AND 2. On full opening, the intermediate zone of the disc is located anterior to the condylar head. Note: Maximum assisted opening of < 40 mm is determined clinically.
	Disc displacement without reduction without limited opening
Description	An intracapsular biomechanical disorder involving the condyle-disc complex. In the closed mouth position, the disc is in an anterior position relative the condylar head and the disc does not reduce with opening of the mouth. Medial and lateral displacement of the disc may also be present. This disorder is NOT associated with current limited opening.
History	Positive for both of the following in the past: 1. Jaw locked so that the mouth would not open all the way; AND 2. Limitation in jaw opening severe enough to limit jaw opening and interfere with ability to eat.
Exam	Positive for the following: 1. Maximum assisted opening (passive stretch) movement including vertical incisal overlap ≥ 40 mm.
Validity	Without imaging: sensitivity 0.54; specificity 0.79. Imaging is the reference standard for this diagnosis.
Imaging	When this diagnosis needs to be confirmed, TMJ MRI criteria2 are the same as for disc displacement without reduction with limited opening. Note: Maximum assisted opening of ≥ 40 mm is determined clinically.
	Degenerative joint disease
Description	A degenerative disorder involving the joint characterized by deterioration of articular tissue with concomitant osseous changes in the condyle and/or articular eminence.
History	Positive for at least one of the following: 1. In the last 30 days,** any TMJ noise(s) present with jaw movement or function; OR 2. Patient report of any noise present during the exam.
Exam	Positive for the following: 1. Crepitus detected with palpation during at least one of the following: opening, closing, right or left lateral, or protrusive movement(s).
Validity	Without imaging: sensitivity 0.55; specificity 0.61. Imaging is the reference standard for this diagnosis.
Imaging	When this diagnosis needs to be confirmed, then TMJ CT criteria106 are positive for at least one of the following: Subchondral cyst(s), erosion(s), generalized sclerosis, or osteophyte(s). Note: Flattening and/or cortical sclerosis are considered indeterminant findings for degenerative joint disease (DJD) and may represent normal variation, aging, remodelling, or a precursor to frank DJD.
	Subluxation
Description	A hypermobility disorder involving the disc-condyle complex and the articular eminence: In the open mouth position, the disc-condyle complex is positioned anterior to the articular eminence and is unable to return to a normal closed mouth position without a manipulative maneuver. The duration of dislocation may be momentary or prolonged. When the patient can reduce the dislocation himself/herself, this is referred to as subluxation. When the patient needs the assistance of the clinician to reduce the dislocation and normalize jaw movement, this is referred to as luxation. This disorder is also referred to as “open lock.” The sensitivity and specificity have been established for only subluxation.
History	Positive for both of the following: 1. In last 30 days,** jaw locking or catching in a wide open mouth position, even for a moment, so could not close from the wide-open position; AND 2. Inability to close the mouth from a wide-open position without a self-maneuver.
Exam	Although no exam findings are required, when this disorder is present clinically, examination is positive for inability to return to a normal closed mouth position without the patient performing a manipulative maneuver.
Validity	Without imaging and based only on history: sensitivity 0.98; specificity 1.00.
Imaging	When this diagnosis needs to be confirmed, imaging criteria are positive for the condyle positioned beyond the height of the articular eminence with the patient unable to close his/her mouth.

TMD diagnosis has always relied on a comprehensive history and clinical exam, with imaging, ionising or otherwise, playing a limited role. There is inadequate evidence to support the use of electronic instruments such as jaw tracking, vibratography, and sonography as diagnostic tools. Imaging of the temporomandibular complex has limitations as well, primarily due to false positives: condylar shape and osseous changes on plain radiographs can occur in asymptomatic individuals and have little bearing on treatment; however, asymptomatic individuals have been shown to have disc displacements or joint effusions on MRI. Although cone-beam and computed tomography have been proven to have high sensitivity and specificity for osseous alterations, evidence-based indications for their usage have yet to be established. Ultrasound is now being utilised to image the temporomandibular complex and has different sensitivity and specificity; nevertheless, imaging within the spectrum of TMDs is probably best used as a complement to clinical diagnosis rather than a definitive diagnostic tool.

Orthodontic management

Over a wide range of modalities, a wide variety of treatments have been offered for TMD. The doctor should demand sufficient scientific evidence to support a treatment's use to confidently choose an appropriate course of action [3]. The following types can broadly be used to categorise all TMD treatment options: 1) Definitive therapy 2) Supportive therapy 3) Conservative therapy.

The goal of definitive treatment is to completely remove or change the etiologic elements that are the cause of the condition. For example, the correct relationship between the condyle and the disc will be set up when the final treatment for anterior disc dislocation of the articular disc is done. TMD therapy employs a wide range of procedures and requires a precise diagnosis. A lot of scientific data should be needed for the clinician to choose a treatment that is likely to be effective.

Reversible Occlusal Therapy

Modifying the patient's occlusal circumstances The best way to reach this goal is to use an occlusal appliance, which is an acrylic device worn over the teeth of one arch and has a surface on the other side that sets and changes the position of the mandible and the way teeth touch. When treating para-functional activity, the appliance offers a mandibular position and occlusion that meet the standards for ideal occlusal relationships. When this appliance is used, an occlusal contact pattern is set up that works with the patient's ideal relationship between the condyle disc and the fossa [2]. A parafunctional state is a warning.

Stabilization Appliance

Typically manufactured for maxillary arches, this offers the patient what is thought to be the best possible occlusal connection. It also prevents canine displacement of the back teeth during eccentric movement. When it is in place, the condyles are in their most musculoskeletal stable position when the teeth are contacting uniformly and concurrently. To remove any orthopaedic instability between the joint position and occlusal position, which is a contributing component in TMD, stabilisation is used as a treatment. For the treatment of problems including muscle discomfort, Studies have shown that it can cut down on the non-functional activity that often happens when people are stressed [11,12].

Requirement for an Anterior Positioning Aid

It is an interocclusal device that stimulates the mandible to move closer to the intercuspal position than usual. Because of the condyle's anterior positioning, which may aid in improving the condyle-disc interaction and provide the tissue with a greater chance to adapt or heal, it may be helpful for the management of some disc derangement illnesses. The primary use of this medication is to reduce disc displacements and disc dislocations. It occasionally helps patients with joint noises [13].

Anterior Bite Planes 

Theseare strong acrylic appliances that are worn over the maxillary teeth and only make contact with the mandibular antecedent teeth. Its primary goal is to disconnect the back teeth and so remove their influence on how the masticatory system works. Indications: It is recommended for the short-term therapy of muscular diseases linked to orthopaedic instability or acute changes in occlusal condition, as well as para-functional activity [14].

Posterior Bite Planes

These comprise hard acrylic sections placed over the back teeth and joined by a cast metal lingual bar. It is often created for the mandibular teeth. The purpose of treatment is to produce a significant change in the vertical dimension and mandibular placement. Indication: It may be beneficial in some disc derangement disorders when there is a severe loss of vertical dimension or when the anterior location of the mandible needs to be altered [15].

Soft or Resilient Appliances

A device made of resilient material, the soft appliance is typically suited to the maxillary teeth. The opposing teeth should be in constant, even contact with one another during treatment. a para-functional activity as they alleviate part of the significant loading pressures experienced during the parafunctional activity [16,17].

Conservative Orthodontic Treatment for TMD

A correct diagnosis of TMJ joint discomfort and dysfunction will result from complete knowledge of the TMJ condition. Soft oral splints are a successful kind of treatment for these patients. This is simple to make and provides the sufferer with excellent comfort. It has been demonstrated that a soft splint made of a 2 mm bioplast can successfully treat TMD with disc dislocation without reducing joint discomfort and permanently heal the problem. aids in mouth opening as well as the anterior bite plane, which is used to further rectify the deep bite [18,19].

## Conclusions

TMD affects a large percentage of people, but the symptoms are rarely debilitating or life-threatening. The severity of TMD in a patient can be determined with the aid of indices, and the source of the problem can be isolated. Distinguishing between mild, moderate, and severe cases of TMD is useful for selecting the most appropriate treatment strategy. The goal of definitive treatment is to completely remove or change the etiologic elements that are the cause of the condition. In order to provide effective care, the doctor needs to first identify and understand the root of the problem.
